# Comprehensive and Systematic Analysis of Gene Expression Patterns Associated with Body Mass Index

**DOI:** 10.1038/s41598-019-43881-5

**Published:** 2019-05-15

**Authors:** Paule V. Joseph, Rosario B. Jaime-Lara, Yupeng Wang, Lichen Xiang, Wendy A. Henderson

**Affiliations:** 1 Sensory Science and Metabolism Unit, Biobehavioral Branch, Division of Intramural Research, National Institute of Nursing Research, National Institutes of Health, Department of Health and Human Services, Bethesda, MD 20892 USA; 2 Digestive Disorders Unit, Biobehavioral Branch, Division of Intramural Research, National Institute of Nursing Research, National Institutes of Health, Department of Health and Human Services, Bethesda, MD 20892 USA; 3 BDX Research & Consulting LLC, Herndon, VA 20171 USA

**Keywords:** Genetics, Biomarkers

## Abstract

Both genetic and environmental factors are suggested to influence overweight and obesity risks. Although individual loci and genes have been frequently shown to be associated with body mass index (BMI), the overall interaction of these genes and their role in BMI remains underexplored. Data were collected in 90 healthy, predominately Caucasian participants (51% female) with a mean age of 26.00 ± 9.02 years. Whole blood samples were assayed by Affymetrix GeneChip Human Genome U133 Plus 2.0 Array. We integrated and analyzed the clinical and microarray gene expression data from those individuals to understand various systematic gene expression patterns underlying BMI. Conventional differential expression analysis identified seven genes *RBM20, SEPT*12, *AX748233, SLC30A3, WTIP, CASP10*, and *OR12D3* associated with BMI. Weight gene co-expression network analysis among 4,647 expressed genes identified two gene modules associated with BMI. These two modules, with different extents of gene connectivity, are enriched for catabolic and muscle system processes respectively, and tend to be regulated by zinc finger transcription factors. A total of 246 hub genes were converted to non-hub genes, and 286 non-hub genes were converted to hub genes between normal and overweight individuals, revealing the network dynamics underlying BMI. A total of 28 three-way gene interactions were identified, suggesting the existence of high-order gene expression patterns underlying BMI. Our study demonstrated a variety of systematic gene expression patterns associated with BMI and thus provided novel understanding regarding the genetic factors for overweight and obesity risks on system levels.

## Introduction

Obesity, defined as a body mass index (BMI) greater than 30 kg/m^2^, is an increasingly prevalent public health concern worldwide. Obesity is associated with several health problems including diabetes, cardiovascular disease, and cancer that increase morbidity and mortality^1–3^. Thus, thousands of studies have been conducted to identify the biological mechanisms underlying obesity in order to identify potential causes of the disease and find methods to mitigate its burden.

Genetics methods exploring biological mechanisms of obesity have utilized multiple molecular methods/techniques including single-gene mutations, transgenic and knockout mouse models, quantitative trait loci (QTL) mapping, association mapping, and gene expression signatures^4–7^. Recently, several genome-wide association studies (GWAS) have identified single-nucleotide polymorphisms (SNPs) associated with obesity^4,8–11^, among which 97 genetic loci were associated with BMI and 49 loci were associated when waist-to-hip ratio was adjusted for BMI^8,10^. Although these studies have helped identify individual mutations and loci associated with BMI, the relevant biological function of these genetic variants is not fully understood.

Leveraging regulatory relationships in system-level gene expression data can highlight biological pathways implicated in obesity and offer further insights into the biological function of these genetic variants. Recent studies have demonstrated that whole-blood transcriptome profiles are valid biological data for weight status and thus provide a more parsimonious method to investigate the functional genomics of obesity, wherein both genes and pathways are included^12–15^. To this end, we generated and integrated gene expression data of venous blood samples and clinical data for 90 individuals. As a convention and validation, we utilized differential expression analysis to detect individual genes responsible for obesity risk. Then, we studied system-level gene expression patterns. We used Weighted Gene Co-expression Network Analysis (WGCNA) to detect gene modules (groups of co-expressed genes) that could potentially interact to mediate increases in weight. We utilized gene connectivity analysis to understand network property changes associated with overweight risks. Lastly, we applied three-way gene interaction analysis to depict the sophisticated gene relationships that may contribute to increased weight. Together this study provides a better understanding of genetic factors in overweight and obese phenotypes through the study of networks, pathways, interactions, and regulation of weight-related genes.

## Materials and Methods

### Design and setting

The study was approved by the Institutional Review Board and the Office of Human Subjects Research at the National Institutes of Health (NIH). Data from participants in a natural history protocol (Clinicaltrial.gov #NCT00824941) conducted at the Hatfield Clinical Research Center, NIH were included. All research activities were performed in accordance with relevant guidelines and regulations. The exclusion criteria of the parent outpatient study included individuals with any known organic disease (e.g., endocrine, gastrointestinal, pulmonary, renal, neurologic, or gynecological pathology). Underweight participants were also excluded. Normal weight, overweight, and obese participants were eligible for inclusion in the study. Participants provided written informed consent during outpatient visits from February 2009 to July 2017. Anthropometric measures (height and weight) and blood samples were collected from fasting participants during the same visit.

### Sample demographics and clinical variables

This study included healthy participants (n = 90) from the parent study (predominately Caucasian, 51% female, mean age 26.00 ± 9.02 years). Detailed baseline demographic characteristics were noted previously^16^. In this study, we divided participants into three genetic-ancestry categories: Caucasian (n = 46), African American (n = 23) and other (n = 21), where Asian and other ethnic groups were categorized as “other”.

Clinical data were collected from the Clinical Research Information System. Weight was measured in triplicate and then averaged; height was measured in duplicate and then averaged. The average height and weight were used to determine BMI, calculated as weight in kilograms divided by height in meters squared. A trained registered nurse completed whole-body air displacement plethysmography (BOD POD^TM^), which determines body fat percent, on all patients. Intra-abdominal height, a measure from the highest abdominal point to the spine in centimeters while the patient is supine, was calculated via ultrasound. Intra-abdominal height was measured without applying pressure to the ultrasound probe. Venous blood samples were sent to the Department of Laboratory Medicine for evaluation of routine cardiovascular laboratory values including fasting glucose, total cholesterol, triglycerides, high density lipoprotein (HDL), low density lipoprotein (LDL), and serum insulin. There were a total of nine missing values in the clinical trait data. Missing values were excluded from related statistics.

### Classification of weight status

We classified weight-status for each person as “normal” weight or “overweight.” We found that the three ethnic groups of this study had very different median BMIs (Caucasian: 24.83; African American: 29.51; and other: 23.46). If we used the conventional criteria of BMI greater than 25 to define overweight, population structures were very different between normal and overweight groups (Supplementary Fig. S1), which could cause differential expression analysis to be confounded by ethnic differences. Thus, we used ethnic-specific BMI cutoffs, which were the median BMI of each ethnic group, to define overweight. The cohort was thus divided into 43 normal weight and 41 overweight individuals that had very similar population structures (Supplementary Fig. S1). Table 1 shows comparisons of demographic and clinical characteristics between normal weight and overweight individuals.

**Table 1:** Comparison of clinical characteristics between normal weight and overweight individuals.

Clinical parameter	Normal weight		Overweight		FDR-adjusted *P*-value
	Mean	Standard deviation	Mean	Standard deviation	
Height	168.58	8.42	172.64	10.11	0.0945
Weight	63.60	9.66	91.33	20.83	2.01 × 10^−9^
BMI	22.32	2.50	30.53	5.96	1.28 × 10^−9^
Body fat	23.22	9.16	32.68	10.85	2.92 × 10^−4^
Cortisol	11.49	5.03	9.49	3.51	0.0787
Insulin	5.87	3.53	10.36	11.13	0.0456
Glucose	86.77	12.91	89.12	8.33	0.375
Cholesterol	157.16	29.46	173.17	25.32	0.0271
Triglycerides	82.28	40.06	106.66	80.96	0.154
HDL	53.72	10.59	50.98	13.41	0.374
LDL	87.05	24.15	102.33	26.14	0.0271
CRP	1.63	2.34	2.73	3.81	0.188
ESR	7.57	6.51	9.93	8.55	0.242
HgA1C	5.30	0.35	5.38	0.35	0.374
IgG	1209.81	263.07	1174.15	217.92	0.525
IgA	193.09	68.24	215.56	81.14	0.244
IgM	112.84	53.03	107.20	57.76	0.643
IgE	169.63	355.64	388.70	1529.08	0.415
Intra-abdominal fat	8.35	1.53	10.96	2.37	2.01 × 10^−6^
Systolic	115.86	13.13	123.15	11.52	0.0271
Diastolic	69.49	6.48	74.07	11.93	0.0784

### RNA isolation, amplification, microarray data processing, and annotation

Details on methods were published in detail previously^16^. In short, blood samples were collected in PAXgene^TM^ RNA (Qiagen, Valencia, CA). Total RNA was extracted and purified using an RNA PAXgene kit (Qiagen, Valencia, CA). Samples in which total RNA passed quality control criteria were used for microarray. All samples were assayed by Affymetrix GeneChip Human Genome U133 Plus 2.0 Array. Quality control and data preprocessing were performed as previously noted^16^. Then, microarray expression data were normalized using quantile normalization. Principal Component Analysis (PCA) indicated that batch effects existed between the two batches (Supplementary Fig. S2). Therefore, the Combat software^17^ was implemented to remove the batch effects in the microarray expression data. PCA indicated that batch effects had been successfully removed (Supplementary Fig. S2).

### Sample size estimation

The sample size needed to perform differential expression analysis was evaluated using the SSPA Bioconductor package^18,19^. Twenty samples (10 normal and 10 overweight samples) were randomly selected from the cohort to estimate power versus sample size.

### Differential expression analysis

Differential expression analysis between normal weight and overweight individuals was performed using the Limma Bioconductor package^20^.

### Evaluation of expressed genes

A total of 13,276 genes were ranked according to their average expression levels. Top ranked genes were more likely to be expressed. According to a study on gene expression profiles, over 91 human and mouse tissues, 30~40% genes, were estimated to be expressed in any tissue^21^. In this study, we assumed that 35% (4,647) genes were expressed.

### Constructing gene co-expression networks

Gene co-expression networks were constructed using the WGCNA package^22^. The 4,647 expressed genes over the 84 individuals were included in the analysis. The scale-free property of the network was visualized (Supplementary Fig. S3). We chose a soft power of 6, which was the lowest power for which the scale-free topology fit index curve flattens out upon reaching a high value. Then, one-step network construction and module detection were performed, with the following parameters: TOMType = “unsigned”, minModuleSize = 10, reassign Threshold = 0, and mergeCutHeight = 0.25. The hierarchical clustering dendrogram was then successfully generated (Supplementary Fig. S4).

### Computing gene connectivity

Topology similarity^23^ was used to measure the similarity between gene expression profiles. We used the WGCNA package to generate the topological matrix for the 4,647 expressed genes. To compute within-module gene connectivity, 99% percentile of the topology overlap matrix^11^ based on the 4,647 expressed genes was computed and used as the cutoff for determining whether two genes are connected. Note that within-module gene connectivity reflects how a gene is connected to other genes within the module, while it is possible that that gene is also connected to several external genes.

To compute gene connectivity changes between normal and overweight gene networks, we first computed TOMs based on the 4,647 expressed genes within normal and overweight gene networks respectively. Then, the 99% percentile of the TOM was computed in each network and used as the cutoff to define gene connection in that network. All 4,647 expressed genes were included to compute gene connectivity (connection to itself was excluded). Assuming that a gene’s connectivity is *C*_*1*_ in network 1 and *C*_*2*_ in network 2, change of gene connectivity between the two networks is defined as:

$${\rm{\Delta }}C=\frac{{C}_{2}-{C}_{1}}{{C}_{2}+{C}_{1}+s}$$

where *s* is a pseudo count of gene connectivity, which is 45.47, the average gene connectivity in this study.

### Functional properties of BMI-associated modules

Functional enrichment analysis was performed using the GOStats Bioconductor package^24^. Only over-representation was assessed via the hypergeometric test. To evaluate enrichment of differentially expressed genes, all 13,276 genes were used as the genomic background. To evaluate enrichment of co-expression gene modules, the 4,647 expressed genes were used as the genomic background. P-values were adjusted by the BH approach^25^.

Canonical pathway analyses were generated through the use of IPA (QIAGEN Inc., https://www.qiagenbioinformatics.com/products/ingenuity-pathway-analysis)^26^ on 157 genes within the “black” module and 35 genes within the lightcyan module. These networks received a score based on the number of genes involved in a network. Top networks and their network functions were also analyzed for each module. As a complimentary approach to IPA, we used Search Tool for the Retrieval of Interacting Genes/Proteins (STRING)^27^ to examine predicted protein-protein interactions (PPIs). We inputted the 157 genes in the “black” module and the 35 genes in the lightcyan module (same genes inputted into IPA). The genes within each module were mapped into PPI networks.

### Regulatory motif analysis

Over-represented transcription factor binding sites (TFBS) and TFBS families in DNA sequences of gene co-expression modules were identified using the oPOSSUM-3 web server^28^. The 4,647 expressed genes were used as the background genes. JASPAR core profiles (i.e., all vertebrate profiles) were used, with a minimum specificity of 8 bits. TFBS search parameters were set as: conservation cutoff: 0.60; matrix score threshold: 85%; amount of upstream/downstream sequence: 2000/2000; number of results to return: top 20 results; sorted by Z-score.

### Three-way gene interaction analysis

Three-way gene expression analysis was performed using the xSyn software^29^. The cutoff of tree height was set to 0.02. The 4,647 expressed genes were sorted in descending order of conditional entropy with weight status. The top 100 genes were selected for detection of three-way gene interactions.

### Ethics approval and consent to participate

The protocol was approved by the Institutional Review Board at the National Institutes of Health. Clinicaltrial.gov # NCT00824941.

### Accession numbers

Microarray data can be found on Gene Expression Omnibus (GEO) under accession number GSE109597.

## Results

### Study data

This nested case-controlled analysis included ancestrally diverse (46 Caucasians, 23 African Americans, and 21 from other ancestral groups) participants were recruited to a natural history protocol (Clinicaltrial.gov #NCT00824941) conducted at the National Institutes of Health (NIH) Hatfield Clinical Research Center. Fasting biological samples and questionnaires were collected from outpatient participants.

A total of 21 clinical variables were assessed, including height, weight, BMI, body fat, cortisol level, insulin level, glucose level, cholesterol level, triglycerides level, high-density lipoprotein (HDL), low-density lipoproteins (LDL), level of C-reactive protein (CRP), erythrocyte sedimentation rate (ESR), Hemoglobin A1c (HbA1c), IgG, IgA, IgM, IgE, Intra-Abdominal Fat (IAF), and systolic and diastolic blood pressure. Missing clinical data were imputed. In this study, obesity risk was approximated by Body Mass Index (BMI).

Whole-blood samples of the 90 participants were assayed for genome-wide expression profiles using the Human Genome U133 Plus 2.0 microarray (Affymetrix, Santa Clara, CA). After removing outlier samples (see Methods), we kept 84 samples for analysis. Expression profiles of 13,276 genes were then generated (see Methods). Potential batch effects were assessed and corrected (see Methods). Distributions of gene expression levels indicated that outlier samples did not exist (Supplementary Fig. S5). We further assessed whether microarray data could be significantly affected by gender, geographic ancestry, or age through PCA, and found that the first and second principal components were not significantly correlated with gender, race, or age, while the third principal component was significantly correlated only with gender (Supplementary Fig. S6 and Table S1). This analysis indicates that for most (if not all) of the analyzed genes, gender, race, nor age affect gene expression levels.

### Differential expression analysis

We asked whether there are differential expressed (DE) genes between normal and overweight individuals. To this end, we first assessed whether a total of 84 individuals were sufficient for detecting DE genes. We used randomly selected 20 samples to draw a plot between power and sample size in each group (Fig. S7), which suggested that with 84 samples available, power is expected to be around 0.55. This power was very modest, and thus we decided to use all samples from different races rather than samples from a single ancestral group for the DE analysis. To ensure that potential DE genes reflected different weight phenotypes rather than population structures, 43 normal weight and 41 overweight individuals, with very similar population structures, were distinguished by a special approach relying on genetic-ancestry specific BMI cutoffs (see Methods).

**Figure 1:**
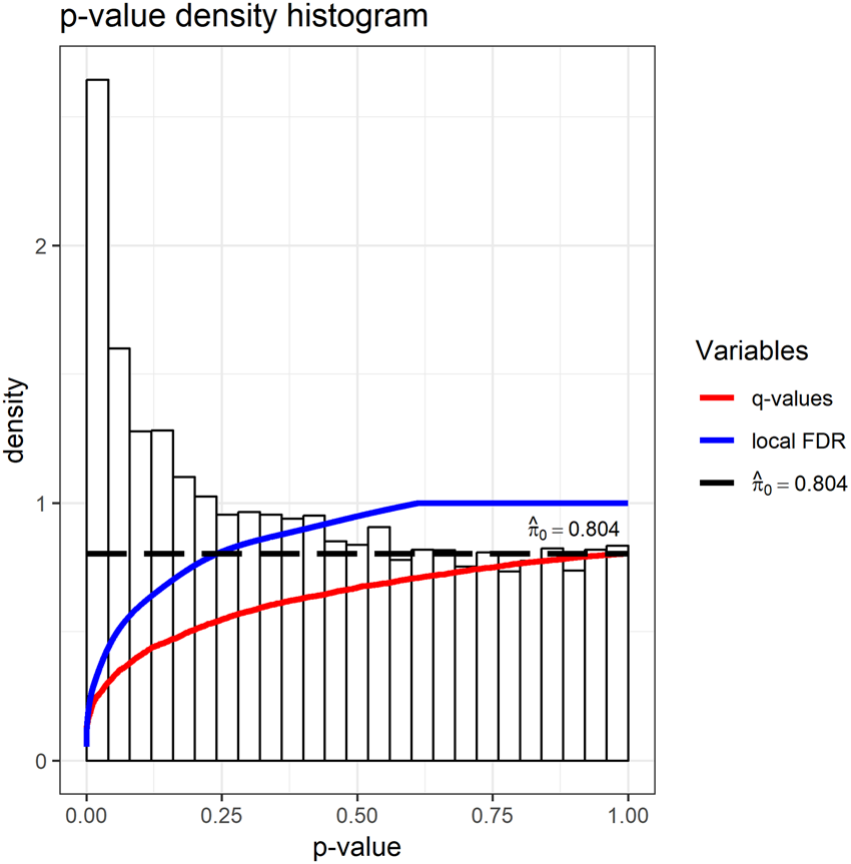
P-value distribution shown with local FDR and q-value levels. P-values were generated by the limma software, and then fed into the qvalue software.

To avoid a decrease in statistical power, we considered that it was not necessary to include gender, race, or age as covariates of DE analysis, because including more variables would reduce power for the same sample size, or more samples would be needed to reach the same power. There may be several genes whose expression levels were associated with gender, race, or age. However, if the overall variables required adjustment, we would expect that they would be significantly associated with the first or second principal component of the microarray data, however, we have shown that none were associated in the previous section (Supplementary Fig. S6 and Table S1). Then, we assessed whether weight status was a major variable affecting the microarray data. We found weight status was not associated with the first or second principal component of the microarray data (Supplementary Fig. S8, p-values: 0.27 or 0.64, *F*-test). Next, we used the q-value software^15^, a more sensitive approach, to assess DE genes between normal and overweight individuals. The default plot of the q-value software (Fig. 1) shows that there is a two-fold density peak within the smallest p-value range (i.e., <0,04), suggesting that there are indeed DE genes. However, the q-value curve goes up quickly in that range, confirming a modest power. Equally speaking, although the number of DE genes was estimated at 13,276 × (1–0.804) = 2602, it was difficult to accurately detect them due to high False Discovery Rate (FDR).

Next, we used the LIMMA software to identify DE genes between normal and overweight individuals. The model matrix defined the weight status as the only factor variable, in addition to the intercept. With a FDR-adjusted p-value cutoff of 0.1, seven genes were found to be differentially expressed, *RBM20, SEPT12, AX748233, SLC30A3, WTIP*, *CASP10*, and *OR12D3* (Table 2). A volcano plot is provided to display significance versus fold-changes (Supplementary Fig. S9).

**Table 2:** Differential expressed genes between normal and overweight individuals.

Gene	Probe_set	Logarithm of fold change	Average expression level	FDR-adjusted p-value
*RBM20*	238763_at	0.457	4.58	7.68 × 10^−2^
*SEP12*	230947_at	−0.150	5.82	7.68 × 10^−2^
*AX748233*	1557267_s_at	0.513	3.90	7.68 × 10^−2^
*SLC30A3*	207035_at	−0.143	6.46	7.68 × 10^−2^
*WTIP*	227411_at	−0.160	6.30	7.68 × 10^−2^
*CASP10*	205467_at	0.155	9.79	7.68 × 10^−2^
*OR12D3*	221431_s_at	−0.145	4.52	8.51 × 10^−2^

Genetic variants of *RBM20* are associated with dilated cardiomyopathy such as heart failure with preserved ejection fraction both in humans and in animal models^30,31^. Mutations of RBM20 gene are known to cause cardiomyopathies because *RBM20* regulates circular RNA production from the Titin (TTN) gene^31,32^. Several studies have documented how individuals with obesity are at higher risk of developing cardiovascular disease^27,33^. This finding suggests that those with obesity may have an increased expression of RBM20 that increases their risk for cardiovascular disease.

*SEPT12* encodes for Septin 12, plays a role including cytokinesis, exocytosis, and membrane dynamics. *SEPT12* is involved in Alzheimer’s disease (AD) related networks, and mutations of this gene have been associated with infertility in men^34,35^. Studies have increasingly pointed to obesity and associated comorbidities as potential contributors to AD pathophysiology, suggesting that higher BMI and obesity are linked to cognitive decline, brain atrophy, reduced white matter and integrity of the blood-brain barrier, and elevated risk for late-onset AD^36^. Likewise, obesity has been shown to impact male fertility function and molecular composition^37^.

*WTIP* is transcribed into Wilms tumor 1 protein (WTIP), which is a scaffold protein that is involved in multiple cellular processes including cytoskeletal organization, cell differentiation, and proliferation^38^. The WTIP molecule plays an important role in kidney-cell remodeling^39^. Modulation of WTIP can lead to the kidney-cell scarring (e.g., focal segmental glomerulosclerosis) that is characteristic in diabetes mellitus^40,41^. Thus, alterations to WTIP may be associated with obesity associated comorbidities, including diabetes^42^.

*CASP10* encodes a protein from the caspase family that is involved in cell apoptosis and survival^43^. Sequential activation of caspases is an important regulation step within the execution phase of cell apoptosis. Alterations in DNA methylation of the *CASP10* gene have been found in pancreatic *B* cells of people with diabetes mellitus^44,45^. Polymorphisms and mutations in this gene are also associated with different types of cancer^46^. This suggests that both genetic and epigenetic modifications of the CASP10 gene may have an important translation implication in diabetes and cancer, which are common comorbid conditions associated with obesity.

Lastly, Olfactory receptor family 12 subfamily D member 3 (*OR12D3*) is a gene that codes for proteins involved in sensory perception of smell. The olfactory receptor proteins are members of a large family of G-protein-coupled receptors (GPCR) arising from single coding-exon genes. Olfactory receptors share transmembrane domain structures with many neurotransmitter and hormone receptors to recognize and G protein-mediated transduce odorant signals^47^. The *OR12D3* gene has been associated with several carcinomas such as stomach cancer, endometrial cancer, and liver cancer^48^. Together these differentially expressed genes encode proteins involved in cellular regulation whose mutations are associated with disease processes that are often associated with obesity, including cancer and cardiovascular disease.

### Weighted Gene Co-expression Network Analysis (WGCNA)

**Figure 2:**
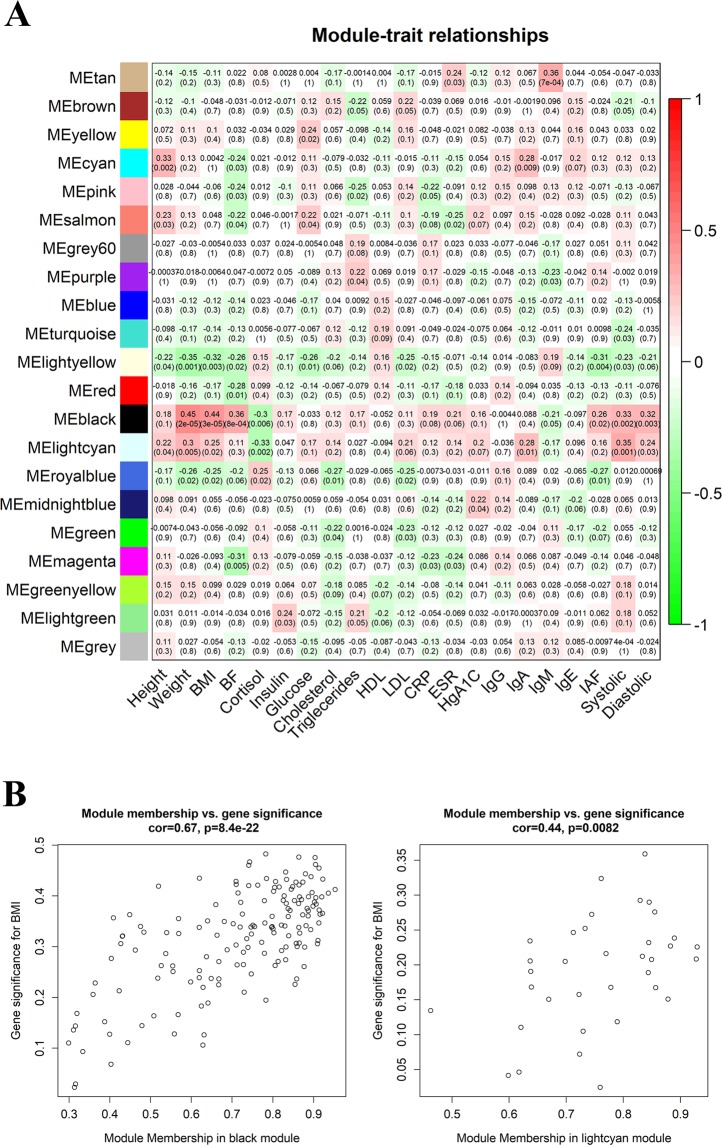
Detection of gene modules associated with clinical traits. (**A**) Matrix of gene module-trait relationships. Each matrix element contains a correlation value between a gene module’s eigenvector (y-axis) and a clinical trait (x-axis) and its corresponding P-value and is colored according to the correlation. Explanation of the traits: BMI: body mass index, BF: body fat, IAF: intra-abdominal fat. (**B**) Module memberships are significantly correlated with gene significance for BMI in “black” and “lightcyan” modules.

Diseases such as obesity can result from dysregulation of gene networks. To more comprehensively understand the gene expression mechanisms underlying weight status, we conducted Weighted Gene Co-expression Network Analysis (WGCNA). To reduce noise in the networks, we selected 35% (4,647) top expressed genes for analysis. The gene co-expression networks were constructed, resulting in 21 gene modules (Fig. 2A). The sizes of these gene modules range from 13 to 1218 genes, with a median size of 103 genes. We further related these gene modules to the 21 clinical variables. Interestingly, we identified two gene modules responsible for the weight, BMI, systolic and diastolic traits - the “black” and “lightcyan” modules (Fig. 2A). The “black” and “lightcyan” modules have 157 and 35 genes respectively. (Supplementary Table S2). We found that in these modules, gene module memberships are highly correlated with gene significance for BMI (Fig. 2B), which enabled us to further prioritize these genes according to gene module memberships (Supplementary Table S2). Note that it appears that the “lightyellow” module is negatively correlated with BMI. However, this module has only 18 genes, and there is no correlation between module memberships and gene significance. Thus, we believed that this negative correlation may not be real.

### Functional properties of BMI-associated modules

Next, we investigated the functional characteristics of these two BMI-associated gene modules. Within the “black” module, we found multiple groups of genes coding for biological functions. The top gene (the most interconnected gene) found in the WGCA “black” module was *BCL2L1* (B-cell lymphoma-2-like protein 1) (Supplementary Table 2). The *BCL2* (B-cell lymphoma-2) family of genes are known to play a major role in apoptosis, and their levels are correlated with BMI and inflammation^49^. The proapoptotic state found in obesity is correlated with insulin signaling, suggesting it can play a role in insulin resistance^50^. Furthermore, the BCL2 family proteins have the potential to affect multiple mechanisms of cardiac damage, including ischemia, calcium dysregulation, and oxidative stress via apoptotic changes^51^. This is consistent with the biological function of genes within the black module (e.g., *BCL2L1, EPOR, BNIP3L, BRAF*, and *GADD45A*). These genes regulate cellular response to stress, and dysregulation of this important biological function may result in the inflammation observed in obesity, diabetes, cardiovascular disease, and cancer^52^. Other groups of genes within the “black” module (e.g., *BCL2L1 SKP1, FBX09, USP12*, and *RNF4*) also participate in cellular catabolism, which if disrupted (as is seen in our high BMI group), can have pathological metabolic sequelae that are observed across the above-mentioned comorbid conditions and cancer.

Another salient group of genes (i.e., *EPOR, BCL2L1, FBXO7, SCNA, APP, PINK1*) within the “black” module have been associated with neuron apoptotic processes. In animal studies, beta-amyloid peptides such as *APP* (amyloid beta precursor protein) was shown to enhance SNCA (∝-synuclein) accumulation leading to neuronal deficits in a transgenic mouse model linking neurodegenerative diseases such as Alzheimer’s disease and Parkinson’s disease^53^. In addition, *FBX07* immunoreactivity in ∝-synuclein have been associated with Parkinson’s disease and multiple system atrophy^54^. A recent study showed that loss of *PINK1* (PTEN-induced putative kinase 1 (*PINK1*), a regulatory protein that is highly expressed in the brain) inhibits apoptosis by upregulating α-synuclein in inflammation-sensitized mouse brains suggesting that loss of *PINK1* may play a novel protective role of inflammation in the brain^55^. There is ample evidence in both animal and human studies demonstrating that obesity is a risk factor for Alzheimer’s Disease, suggesting that the chronic low-grade systemic inflammation seen in obesity leads to neuroinflammation^56–58^. However, the association of Parkinson’s disease-risk and obesity remains debatable^59,60^, warranting more research.

The top gene within the “lightcyan” module was the *SPARC* (secreted protein acid and rich in cysteine) gene. This gene modulates tissue physiology and alters cell-ECM interaction, cell proliferation, and cell migration^61^. Its role in regulating wound healing, angiogenesis, tumorogenesis, and inflammation is consistent with its positive correlation to diabetes and cardiovascular disease^61^. Other genes within the “lightcyan” module, which interact with *SPARC* to modulate wound healing, include *PPKAR2B* (protein kinase cAMP type II subunit beta), *TREML1* (triggering receptor expressed on myeloid cell like 1), and vinculin. Mutations in these genes may also lead to vascular disease, cardiomyopathy, and dementia/neurodegeneration^62,63^, respectively. Thus, both of the most interconnected genes of the “black” and “lightcyan” modules and other genes within these two models are known to play a key role in obesity and other pathogenic processes leading to diabetes, cardiovascular disease, cancer, and neurodegeneration.

We also performed GO biological process and KEGG pathway enrichment analysis using the Gostat software^24^, with a p-value cutoff of 0.01. The results (Supplementary Table S3) show that the “black” module is mainly enriched for catabolic processes and Wnt signaling pathway, while the “lightcyan” module is mainly enriched for muscular system, cell migration/motility and vasculature development. Analysis of top 20 over-representative transcription factor binding sites (Supplementary Table S4) suggested that only four transcription factors – FEV, HLF, RREB1 and ZNF354C – were shared between the two gene modules, however, zinc finger transcription factors are the major family in both gene modules.

**Figure 3:**
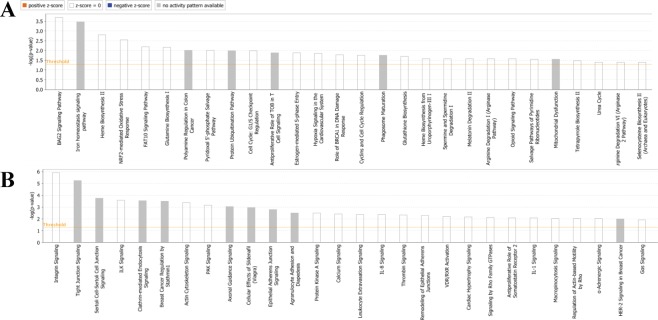
Gene coexpression networks in BMI-associated modules. Every node depicts a gene and lines represent a coexpression relationship. (**A**) Gene coexpression networks of the “black” module. (**B**) Gene coexpression networks of the lightcyan module. The figure was generated through the use of IPA (QIAGEN Inc., https://www.qiagenbio-informatics.com/products/ingenuity-pathway-analysis).

IPA identified significant networks in the “black” and “lightcyan” modules (Fig. 3A,B)^26^. We identified eleven gene networks in the black module, which had scores between 2 to 47. Gene networks in the “black” module had associated network functions including cell morphology, cancer, inflammatory responses, and neurological disease. Top canonical pathways included iron homeostasis, nuclear factor (erythroid-derived 2) NRF2-mediated Oxidative Stress Response, and glutamine biosynthesis. Four gene networks were found in the “lightcyan” module with scores of 40, 19, 14, and 1. Gene networks in the “lightcyan” module were associated with cancer, cell-to-cell signaling, cellular morphology, and smooth muscle. Top canonical pathways included breast cancer regulation endocytosis signaling, integrin signaling, tight-junction signaling, Sertoli cell junction, Integrin-linked kinase signaling, and clathrin-mediated endocytosis signaling.

**Figure 4:**
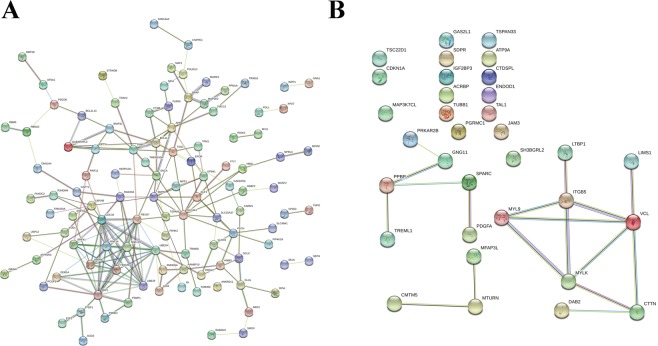
Protein-protein interactions in BMI-associated modules. Each node denotes a gene and each line indicates a protein-protein interaction. (**A**) Protein-protein interactions in the “black” module. (**B**) Protein-protein interactions in the lightcyan module.

The “black” module analysis found 150 modes, 165 edges, and a PPI enrichment value of <1.0e-16. The proteins that were closely associated with other proteins included, RNF41, RNF14, SKP1, UBE2H, and NS FBXO9. These proteins are known to be associated with pathways including regulation mitophagy, TOR signaling, and ubiquitin-dependent catabolic processes (Fig. 4A). The “lightcyan” PPI had 33 nodes, 15 edges, and a PPI enrichment value of 6.6e-05. Proteins with high PPIs included, ITGB5, MYLK, and VCL. These proteins are involved in antigen processing and presentation of exogenous peptides, virus receptor activity, aorta smooth muscle tissue morphogenesis, and platelet aggregation (Fig. 4B).

### Gene connectivity analysis

**Figure 5:**
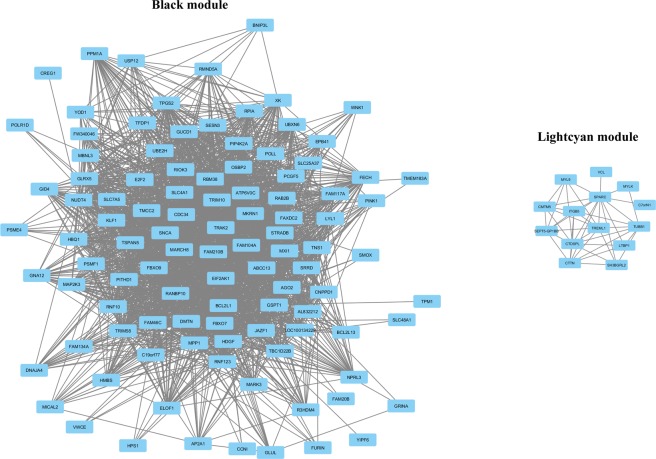
Network visualization of the gene modules associated with weight traits. Each node denotes a gene, and each line indicates that two genes have a topology similarity greater than 0.99 quantile. Note that genes not connected based on our criteria are not displayed.

Gene connectivity measures the number of genes that a gene is connected to. Hub genes are frequently believed to be more functionally important than peripheral genes. Here we adopted a customized approach to determine whether two genes were connected – their topology similarity should be greater than 0.99 quartile of topology overlap matrix (see Methods). We computed the within-module gene connectivity for the “black” and “lightcyan” modules. Visualization of gene connectivity (Fig. 5) suggests that the genes in the “black” module are more inter-connected than the “lightcyan” module. Only 40% of the 35 genes in the “lightcyan” module are interconnected, while 66% are interconnected in the “black” module. We further sorted the genes within these two modules by importance according to gene connectivity (Supplementary Table S5). This ranking is similar to that of the gene module membership.

Next, we evaluated potential gene connectivity changes between normal and overweight individuals. Genes that are converted from hub to peripheral genes or from peripheral genes to hub genes are more likely to perform important roles in development of obesity. Weighted gene co-expression networks within normal and overweight individuals respectively were constructed based on the 4,647 expressed genes. Then, we used 0.99 quantile of topology overlap matrix to define the two genes’ connection in normal and overweight networks respectively. Subsequently, we applied a formula to compute gene connectivity changes (see Methods). We then used gene connectivity change >0.5 to define nonhub genes being converted to hub genes in overweight individuals, and gene connectivity change <−0.5 to define hub genes being converted to nonhub genes in overweight individuals. We identified 246 hub genes that were converted to nonhub genes and 286 nonhub genes that were converted to hub genes in overweight individuals (Supplementary Table S6). However, functional significance analysis indicated that there was no enriched GO biological process term in these genes. This analysis suggests that a considerable number of genes are differentially connected between normal and overweight individuals, shedding some light on the network dynamics underlying human weight.

### Three-way gene interaction analysis

**Figure 6:**
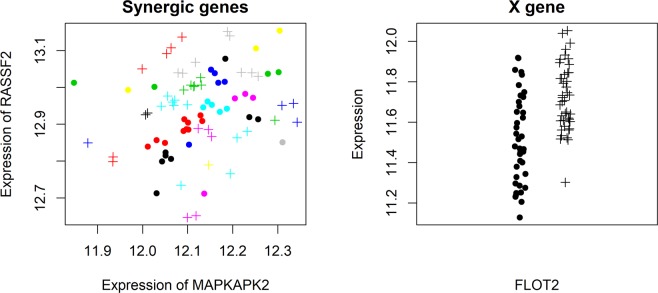
Visualization of a 3-way gene interaction underlying the microarray data. “.” represents downregulation of gene X whereas “**+**” represents upregulation of gene X. Clusters tend to be filled with the same status of gene X, and thus, an optimal synergy is achieved. The gene X shows differential expression between downregulation and upregulation statuses.

We investigated whether there were more sophisticated gene expression patterns underlying human weight. To this end, we considered three-way gene interactions, defined as two gene expression profiles being clustered in different space locations. These highly synergistic genes would be under the control of a third gene (i.e., gene X)^29^ and could have differential expression or outlier differential expression associated with weight status. We utilized the xSyn software for this analysis. We computed the top 100 genes with the highest conditional entropy. We then searched the 100 genes for gene pairs with synergy greater than 0.9. Lastly, we assessed whether any gene pairs associations could be attributed to a third control gene. We identified a total of 28 three-way gene interactions (Supplementary Table S7). We then randomly chose a three-way gene interaction for visualization (Fig. 6). This analysis suggests complicated high-order gene interactions that may contribute to the genetic mechanisms affecting human weight.

## Discussion

Gene expression data can offer key information for understanding the genetic mechanisms underlying increases in body weight and obesity. In this study we identified 7 differentially expressed genes, *RBM20, SEPT12, AX748233, SLC30A3, WTIP, CASP10*, and *OR12D3*. These genes code for proteins that play an important biological function, including cellular apoptosis, cytoskeletal organization, and olfaction. Alteration of these genes can result in pathological processes leading to diabetes, cardiovascular disease, cancer, or dementia. We found that three (AX748233, CASP10, and OR12D3) out of the seven DE genes can be found to be differentially expressed under different weight status in external data (Supplemental Fig. S10). It is well known that the repeatability of microarray experiments is low due to different experimental designs, protocols, population structures, systematic errors, etc.^64^. However, this also suggests that new experiments are still desired to achieve deep understanding of the genetic mechanisms for body weight and obesity. A combination of DE analysis and a variety of network analyses were not performed previously, so this study has provided many new insights.

Genes and protein interactions identified through IPA and STRING also contributed to the investigation of the role of these genes in gene networks and biological processes. Genes and protein networks within the “black” module were involved in cellular regulatory processes, and many were associated with tumor formation and multiple forms of cancer. Further supporting previously identified sub-networks of genes associated with cancer and cellular proliferation through network modelling^65^. Genes in the lightcyan module were associated with cellular and tissue structure, cancer, and cardiovascular biological processes. This suggests that BMI-associated genes closely interact in gene networks that are involved in the pathologic pathways of comorbid conditions (i.e., cancer and cardiovascular disease) that have been linked to obesity. These data are in line with previously discussed functions of differentially expressed genes in overweight/obese individuals. Further research should determine the relationship, directionality, and interaction of these obesity-related genes with cardiovascular disease and cancer-related genes.

Hybrid approaches that incorporate both genetic expression and enrichment analyses offer further insight into the functional significance of genes within complex biological pathways. Previous studies implemented limited analysis approaches. Based on differential expression and enrichment analyses, we found that the expression of seven genes was significantly correlated with body weight, some of which were previously reported in another study^14^. In addition, another study found that “ribosome,” “apoptosis,” and “oxidative phosphorylation” pathways were associated with obesity and were identified as pathway-specific predictors for obesity^66^.

Hybrid approaches with more advanced enrichment analyses provide a more complete understanding of the functional significance of genes. A recent study by implemented hybrid approaches using differential expression and enrichment analyses^13^ reported 32 genes and 3 pathways associated with BMI. They emphasized enrichment of NF-kappa B pathways in the 32 DE genes. However, only 2 (6.3%) DE genes were related to NF-kappa B pathways, and therefore the role of NF-kappa B pathways is not fully understood. Using WGCNA analysis, they identified a module of 68 genes associated with BMI. Those genes were thought to be related to a variety of biological processes, while each biological process contained only two or three genes (≤4.4%).

In the current study, we incorporated a robust hybrid method that identified more relationships between genes and their function within catabolic processes. We found that up to 43 (27.4%) of the 157 genes in the “black” module were related to catabolic processes (compared to ≤4.4% found by Wang *et al*.^13^. Thus, our study offers a more complete understanding of the functional role of differentially expressed genes in metabolic processes. Since it is well established that metabolic processes play a role in increased weight and obesity^10,67,68^, this information is key in identifying potential contributors to overweight and obese phenotypes.

In this study, we demonstrated that complex high-order gene interactions could also play a role in the genetic mechanisms underlying overweight and obese phenotypes. To our knowledge, this is the first paper to show high-order gene interactions in overweight and obese phenotypes. With improvement of computation capacity and development of intelligent approaches, high-order interactions will be investigated more frequently and successfully, providing new avenues toward fully understanding the genetics of increased weight and obesity. Although, a comprehensive analysis of microarray data was conducted in this study, gene expression is only one layer of genetic information. We previously showed that the combination of gene expression and SNP data could generate relatively better outcomes for predicting BMI^16^. Thus, future studies should integrate multiple layers of genetic and epigenetic data to better understand the role of genes and environmental factors in predicting risk for increased weight and obesity.

Future research should also re-examine gene co-expression of BMI-related genes across different tissues. In the current study, blood was collected to extract gene expression data. Although studies suggest there is comparability of genomic information derived from blood samples compared to other tissues, the correspondence between peripheral blood and tissues of interest is not fully understood^69,70^. A recent review of gene-expression across obesity relevant tissues found diverse BMI-related genes across tissues and tissue specific patterns^70^. Thus, studies utilizing animal tissue and human tissue (from existing tissue banks) are needed to examine the gene co-expression patterns identified in this study across other metabolic and obesity-related tissues. This would offer a more complete understanding of the functional role of genes and may help identify target tissues to study and treat obesity.

## Conclusions

In this study, we integrated gene expression and clinical data of 90 healthy individuals to investigate the genetic mechanisms of overweight and obesity. A variety of computational approaches were applied to generate comprehensive and systematic gene expression insights into human weight. We identified seven differentially expressed genes associated with BMI. Using WGCNA, we found two modules significantly associated with BMI. The two modules displayed different connectivity and functional significance features and were frequently regulated by zinc finger family transcription factors. The major module was enriched for catabolic genes, which is consistent with other studies highlighting the roles of metabolic genes in the etiology of overweight and obesity. We also profiled the genes switching hub status between normal and overweight individuals, as well as three-way gene interactions associated with weight status. Our analyses have generated comprehensive and systematic insights into gene expression mechanisms underlying BMI and have highlighted complex high-order interactions that should be further explored in in future studies.

## Supplementary information

Supplementary Material 1

Supplementary Table S2.

Supplementary Table S3.

Supplementary Table S4.

Supplementary Table S5.

Supplementary Table S6.

Supplementary Table S7.

## Data Availability

Dataset available in supplement. Additional data available upon request from senior author hendersw@mail.nih.gov upon request.
