# Supplementary material for: Comprehensive and Systematic Analysis of Gene Expression Patterns Associated with Body Mass Index
Source: Sci Rep. 2019 May 15;9:7447. doi: 10.1038/s41598-019-43881-5 (PMC6520409; doi:10.1038/s41598-019-43881-5)

**Comprehensive and Systematic Analysis of Gene Expression Patterns Associated with Body Mass Index**

Paule V. Joseph^1^, Rosario B. Jaime-Lara^1^, Yupeng Wang^3^, Lichen Xiang^2^, Wendy A. Henderson^2^

^1^ Sensory Science and Metabolism Unit, Biobehavioral Branch, Division of Intramural Research, National Institute of Nursing Research, , National Institutes of Health, Department of Health and Human Services, Bethesda, MD, 20892, USA

^2^ Digestive Disorders Unit, Biobehavioral Branch, Division of Intramural Research, National Institute of Nursing Research, , National Institutes of Health, Department of Health and Human Services, Bethesda, MD, 20892, USA

^3^BDX Research & Consulting LLC, Fairfax, VA 22031, USA

*Corresponding author:

Dr. Wendy A. Henderson, [hendersw@mail.nih.gov](mailto:hendersw@mail.nih.gov), Telephone: +1-3014519534, Fax: +1-3014801413

Fig. S1. Comparison of racial compositions between normal and over weight individuals without (A) and with (B) use of race-specific BMI cutoffs for determining over weight status.


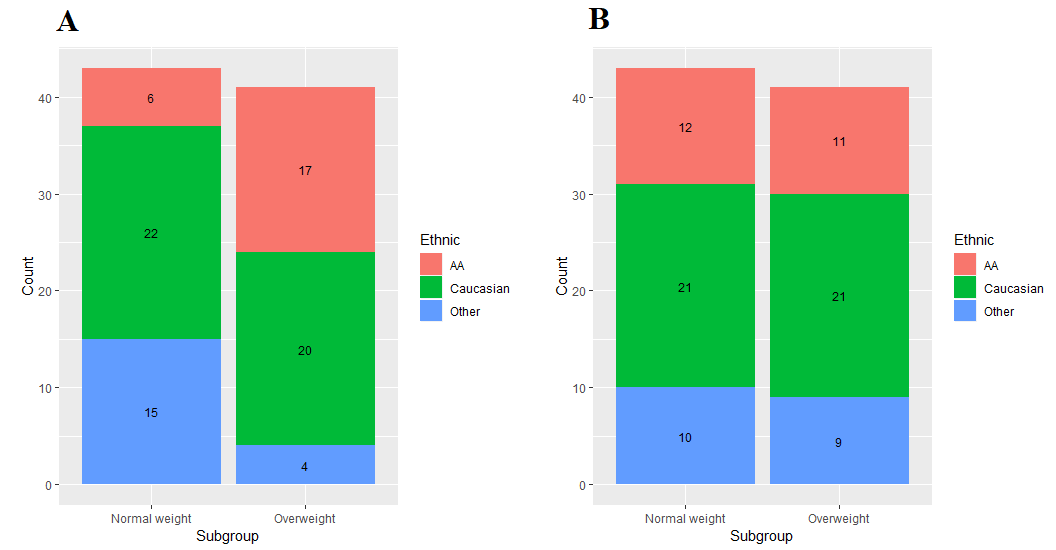


Fig. S2. PCA analysis of microarray data before and after batch effect removal.

**
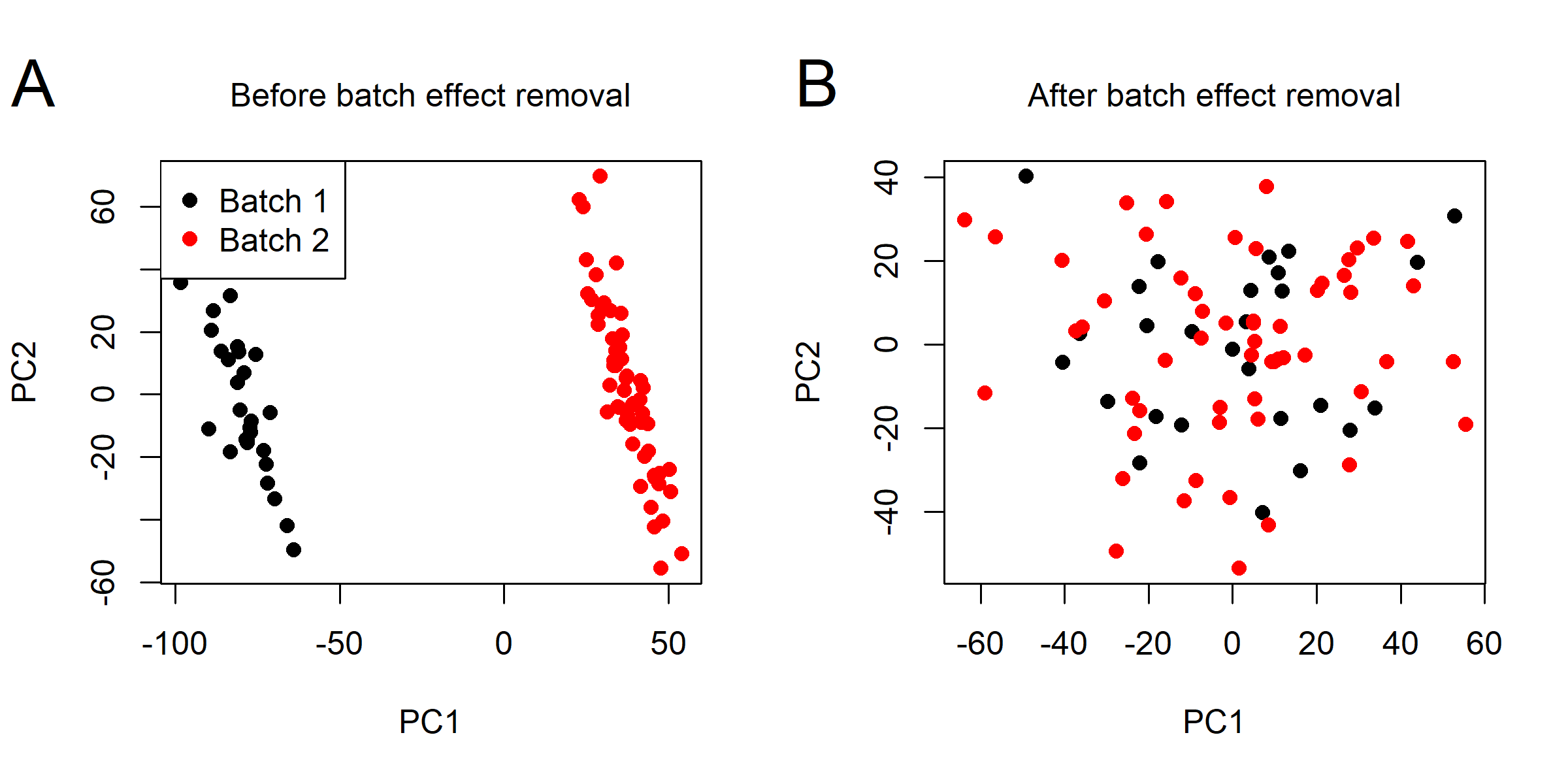
**

Fig. S3. Scale-free topology properties of the generated coexpression network.

**
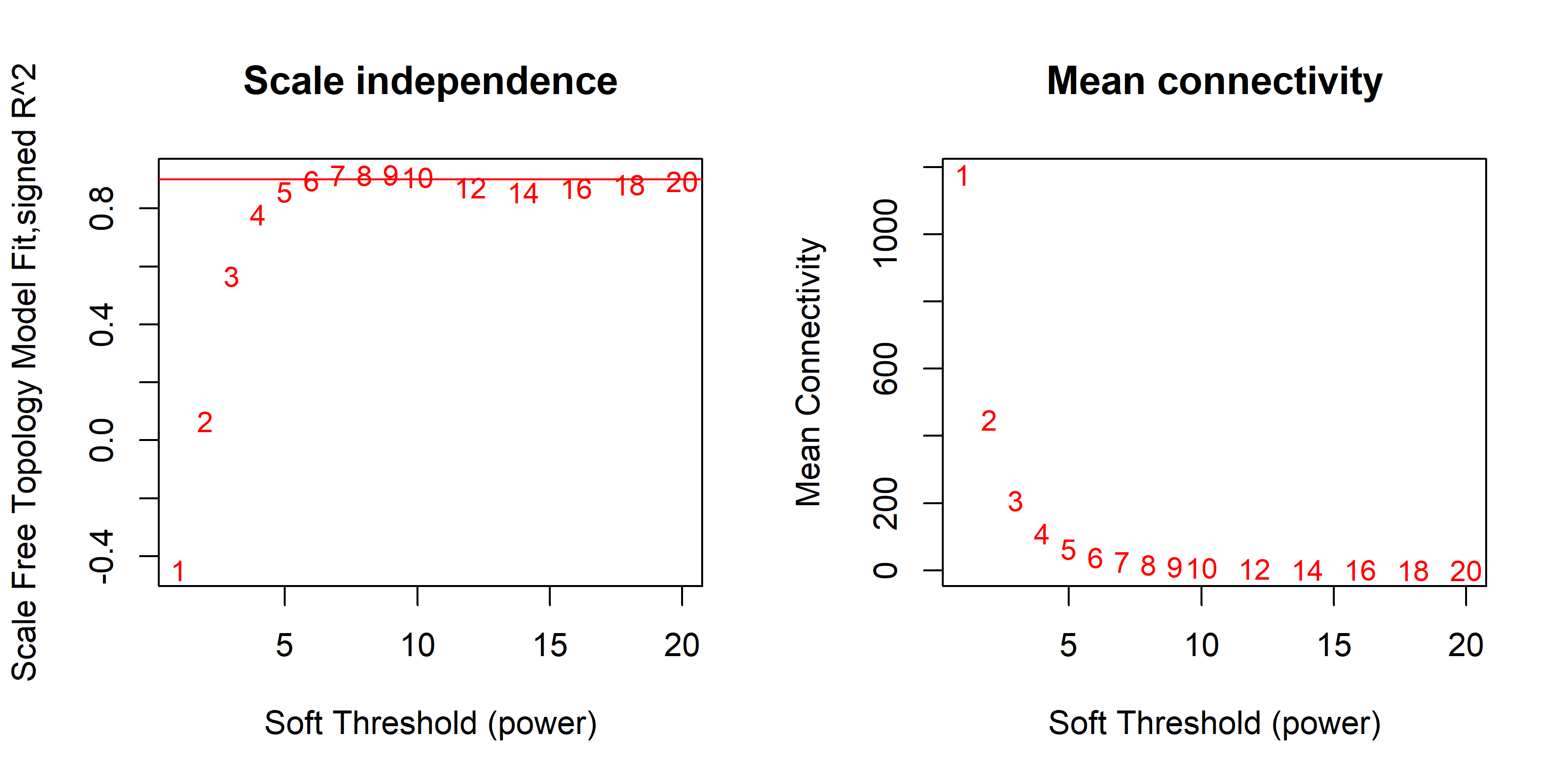
**

Fig. S4. Gene dendrogram and co-expression modules generated by WGCNA and labeled by colors.

**
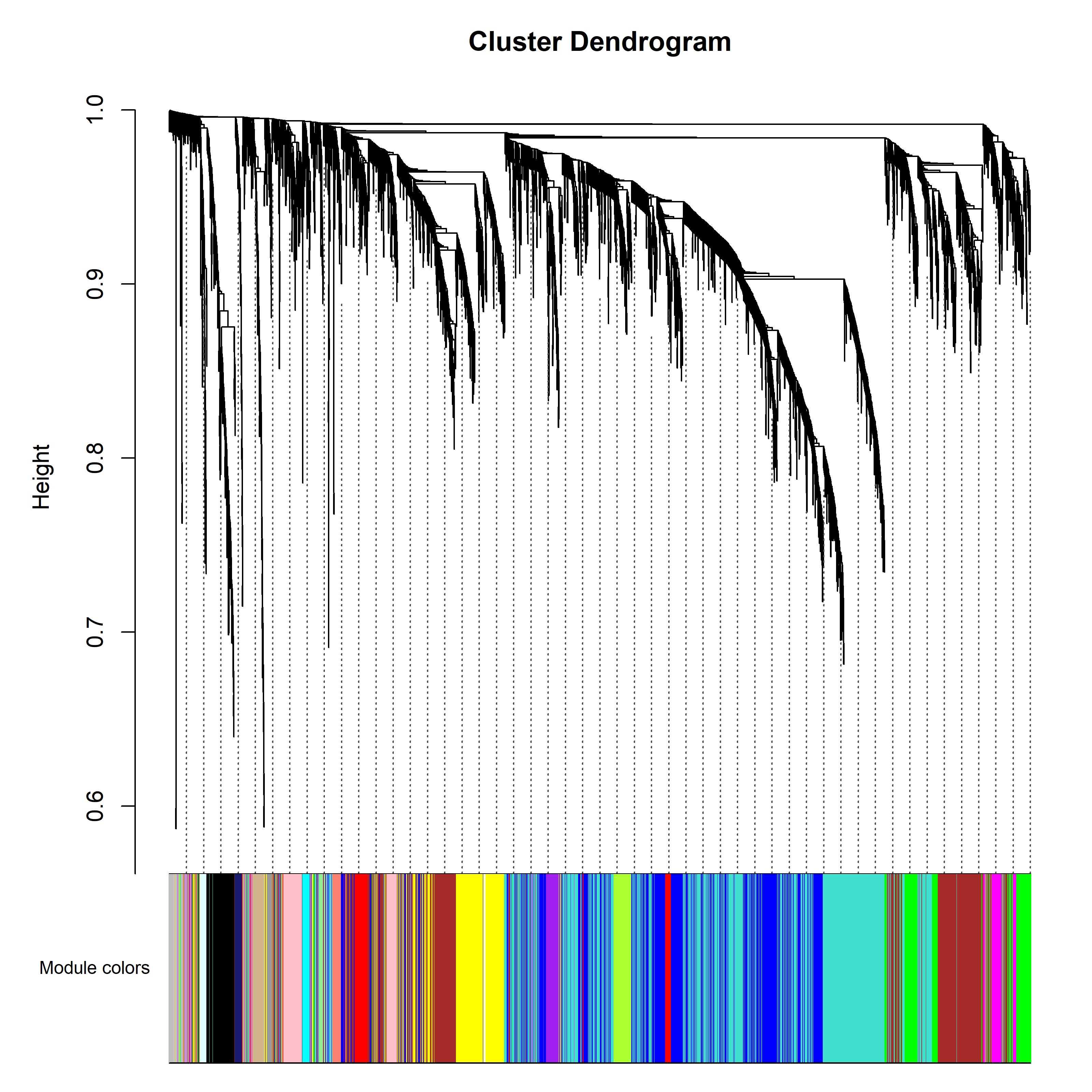
**

Fig. S5. Signal (gene expression level) distributions of microarray data for all individuals after preprocessing

**
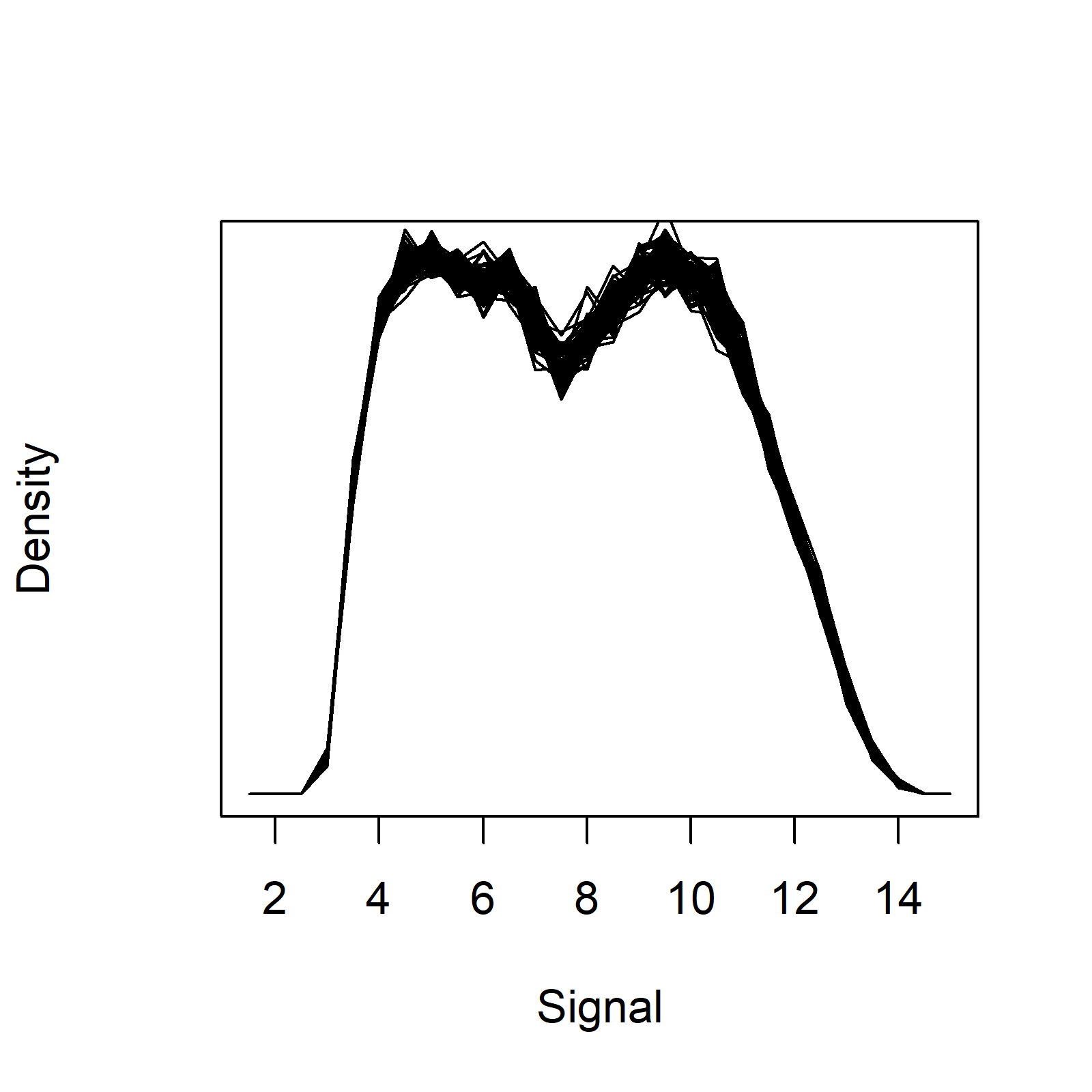
**

Fig. S6. Major principal components of microarray data are not associated with gender, race or age.

**
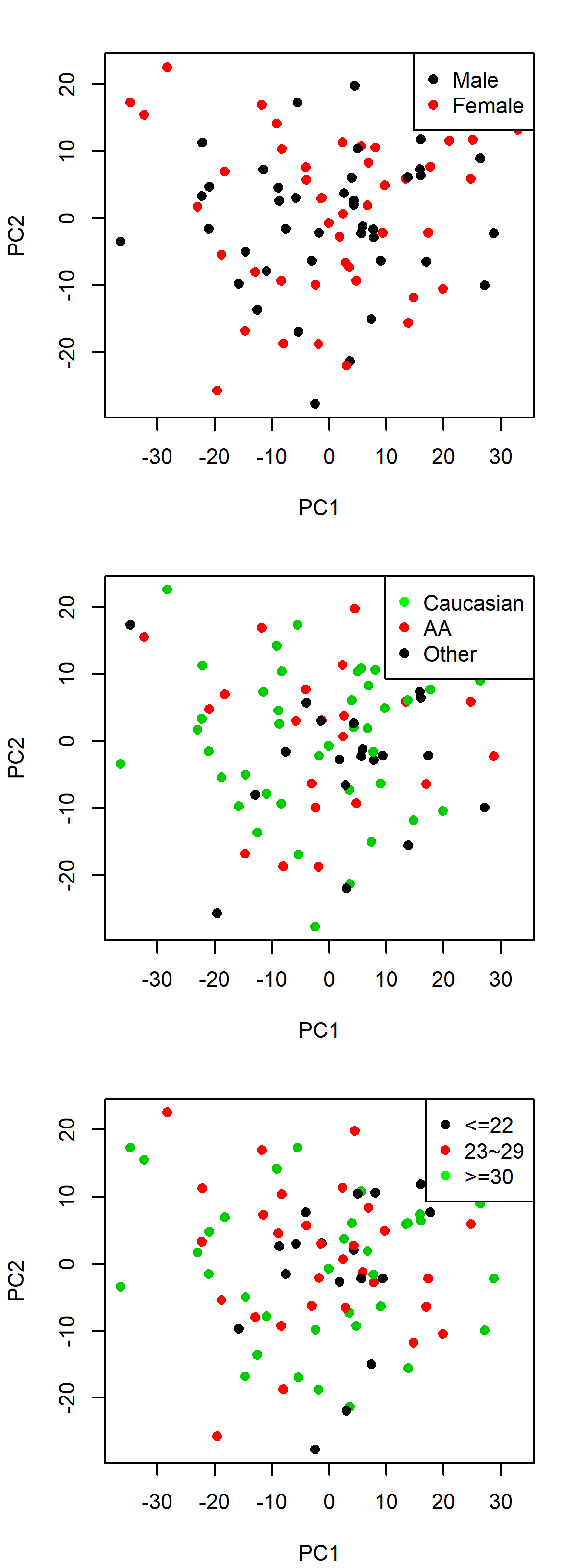
**

Table S1. Microarray data are not significantly associated with gender, race or age by ANOVA.

| **Variable** | **P-value of F test** | | |
| --- | --- | --- | --- |
|  | **PC1** | **PC2** | **PC3** |
| Gender | 0.948 | 0.605 | <2.2×10^-16^ |
| Race | 0.539 | 0.319 | 0.124 |
| Age | 0.652 | 0.493 | 0.134 |

Fig. S7. Power versus sample size based on 20 randomly selected samples.

**
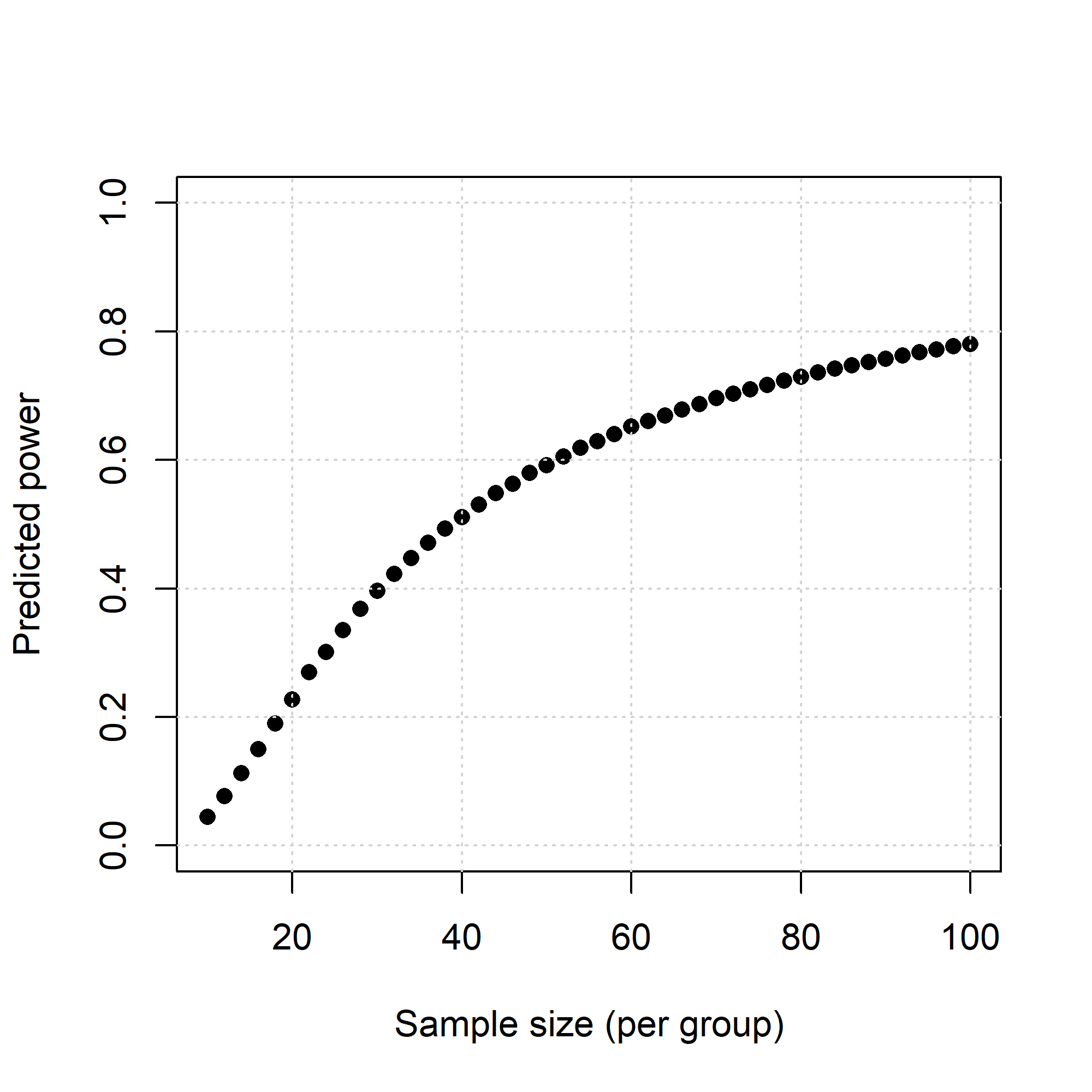
**

Fig. S8. First and second components of microarray data are not associated with weight status.

**
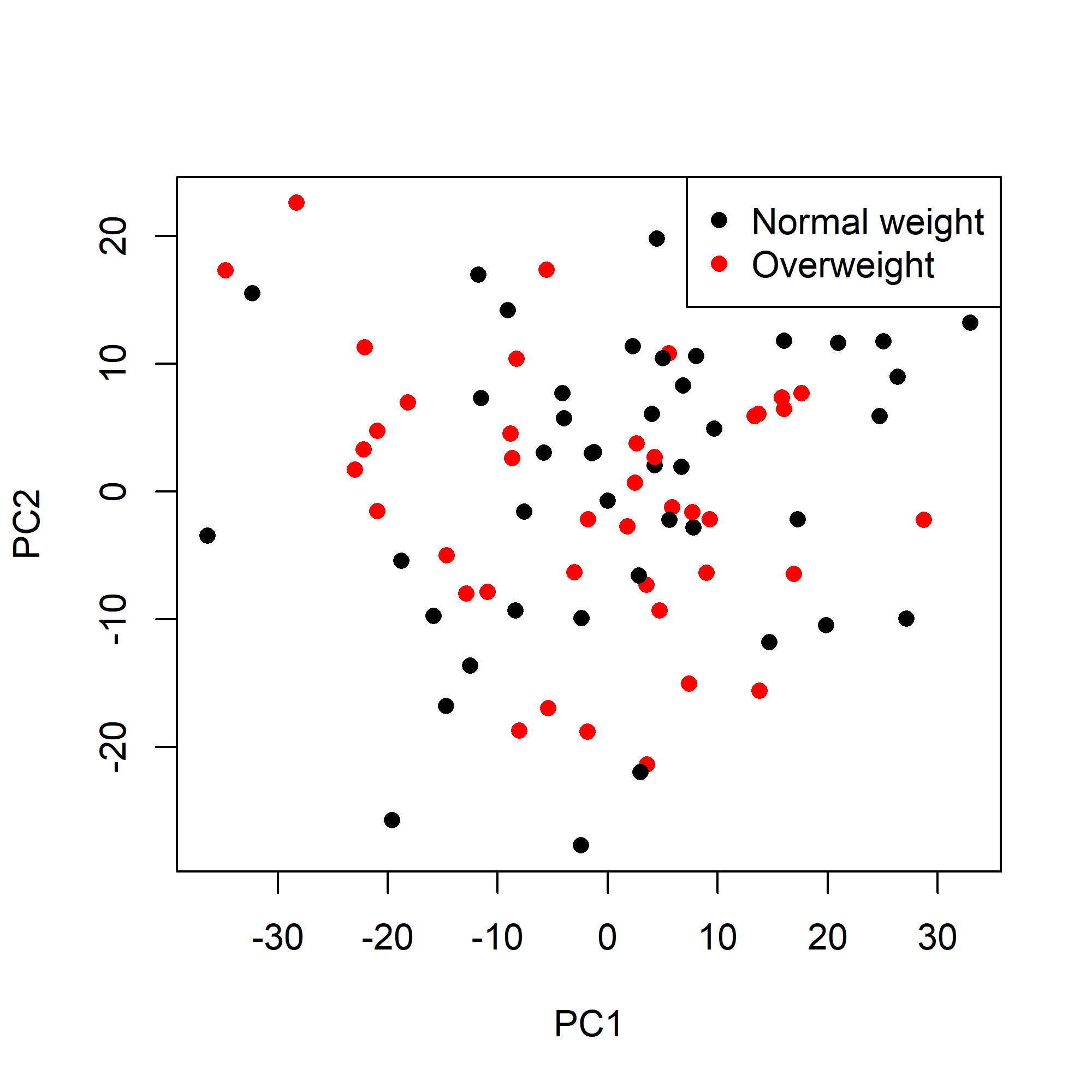
**

Fig. S9. Volcano plot to visualize significance versus fold-change following the DE analysis


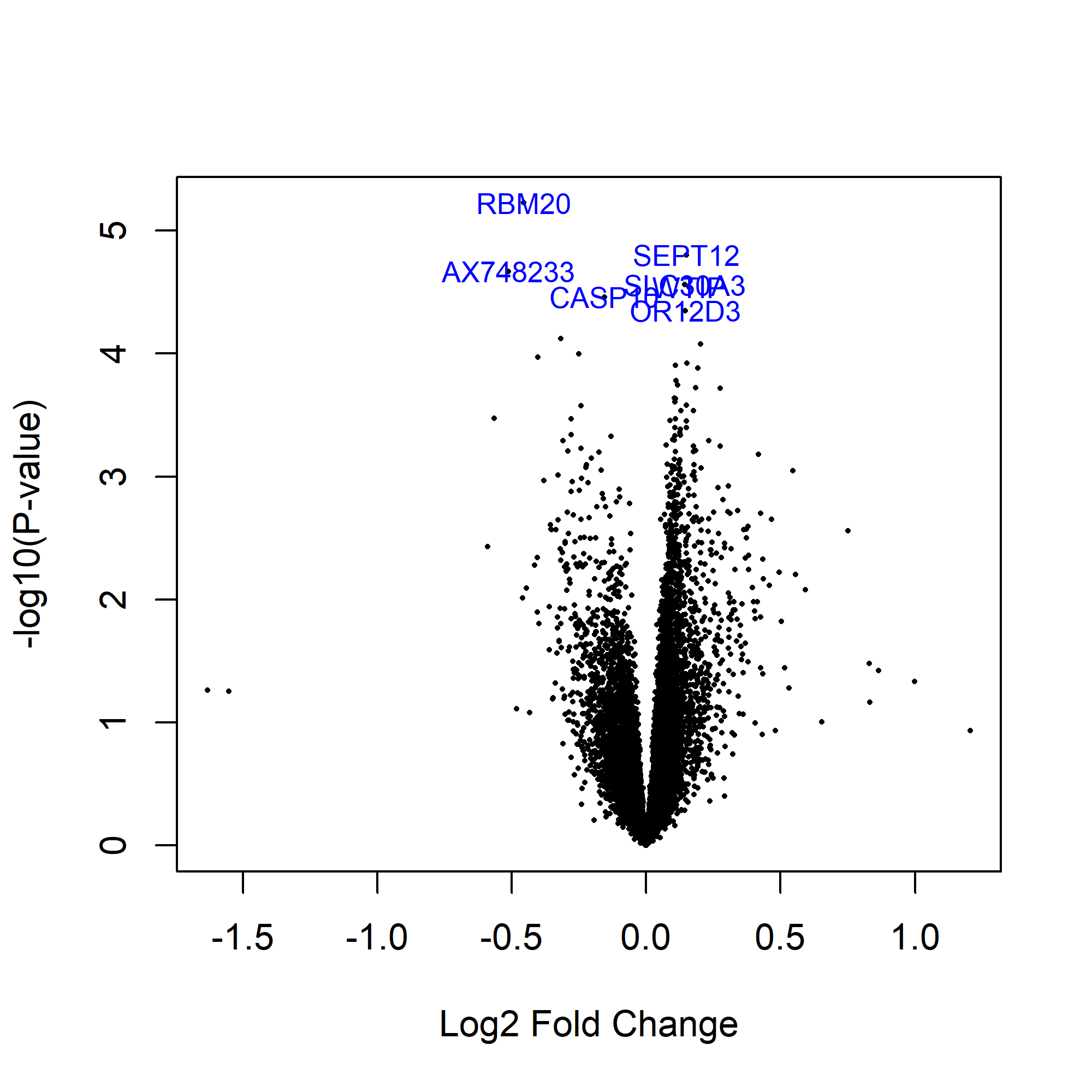


Fig. S10. Three DE genes of this study were found to be differentially expressed in external microarray datasets related to weight traits.


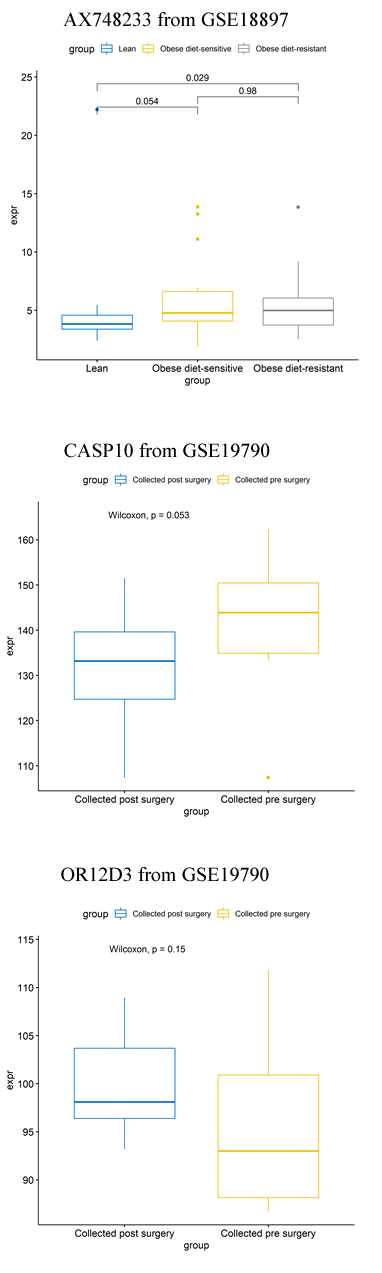

Supplement: Supplementary file 1 — Supplementary Material 1 [file 41598_2019_43881_MOESM1_ESM.docx]
